# Supplementary material for: Quality control in oocytes by p63 is based on a spring-loaded activation mechanism on the molecular and cellular level
Source: eLife. 2016 Mar 14;5:e13909. doi: 10.7554/eLife.13909 (PMC4876613; doi:10.7554/eLife.13909)
Supplement: Supplementary file 1. — Constructs were expressed in rabbit reticulocyte lysate and subjected to size exclusion chromatography on a Superose 6 3.2/300. All constructs formed dimers indicating that none of these residues are involved in essential contacts inside dimeric TAp63α. DOI: http://dx.doi.org/10.7554/eLife.13909.024 [file elife-13909-supp1.docx]

**Supplemental Table 1. Mutagenesis screen of residues on the surface of the DBD.** Constructs were expressed in rabbit reticulocyte lysate and subjected to size exclusion chromatography on a Superose 6 3.2/300. All constructs formed dimers indicating that none of these residues are involved in essential contacts inside dimeric TAp63α.

| **TAp63α _(10-614)_ construct information** |
| --- |
| D131A, Y132A |
| W153A |
| E158A |
| I166A |
| N207A, L210A |
| H208A, E213A, F214A |
| E209A, E238A |
| R212A, E213A, E216A |
| R212A, E213A, F214A, E216A, Q218A, I219A |
| R212A, E213A, E216A, S232A, H233A, Q235A |
| R212A, I219A |
| R212A, I219A, S232A, H233A, Q235A |
| E213A |
| F214A |
| Q218A, I219A |
| Q218A, I219A, S232A, H233A, Q235A |
| P221A, P222A |
| S232A, H233A, Q235A |
| H233A, Q235A, V237A |
| E238A, D239A, I241A |
| V274A |
| V274A, N278A, R279A |
| R279A |
| R279A, P281A, L283A |
| D292A, V295A |
| R304A |
| R311A |
